# Signet-Ring Cell Squamous Cell Carcinoma: A Biphenotypic Neoplasm of the Gastro-Esophageal Junction with Uncertain Biological Potential: Case Report and Literature Review

**DOI:** 10.3390/ijms24119535

**Published:** 2023-05-31

**Authors:** Catalin Bogdan Satala, Zsolt Kovacs, Tivadar Bara, Ioan Jung, Simona Gurzu

**Affiliations:** 1 Department of Pathology, George Emil Palade University of Medicine, Pharmacy, Science and Technology, 540012 Targu Mures, Romania; stlcatalin92@yahoo.com (C.B.S.); kovacska_zsoltkovacs@yahoo.com (Z.K.); jungjanos@studium.ro (I.J.); 2 Department of Pathology, Clinical County Emergency Hospital, 540136 Targu Mures, Romania; 3 Research Center for Oncopathology and Translational Medicine (CCOMT), George Emil Palade University of Medicine, Pharmacy, Science and Technology, 540136 Targu Mures, Romania; 4 Department of General Surgery, Clinical County Emergency Hospital, 540136 Targu Mures, Romania; btibi_ms@yahoo.com

**Keywords:** squamous cell carcinoma, signet-ring cell, gastro-esophageal junction, epithelial-mesenchymal transition, Sox2, CD44

## Abstract

The signet-ring cell variant of squamous cell carcinoma (SCC) is an extremely rare histological subtype, with only 24 cases (including the present case) reported in the Medline database: 15 affecting the external surface of the body, 3 in the lung, 2 affecting the uterine cervix, 1 involving the gingiva, another one affecting the esophagus and the present case that is the first reported at the gastro-esophageal junction (GEJ). In one case, the location of the lesion was not mentioned. A 59-year-old male patient underwent segmental eso-gastrectomy for carcinoma of the GEJ. The microscopic examination showed a pT3N1-staged SCC composed of solid nests admixed in over 30% of the tumor, with cells having eccentrically located nuclei and clear vacuolated cytoplasm. The signet-ring cells did not show mucinous secretion and were positive for keratin 5/6 and vimentin, with nuclear expression of β-catenin and Sox2 and focal membrane positivity for E-cadherin. Based on these features, the case was considered a signet-ring SCC with epithelial–mesenchymal transition. Thirty-one months after surgery, the patient was disease-free, with no local recurrence and no known distant metastases. In SCC, a signet-ring cell component might be an indicator of the dedifferentiation of tumor cells towards a mesenchymal molecular subtype.

## 1. Introduction

According to International Agency for Research on Cancer (IARC) statistics, esophageal/gastro-esophageal junction (GEJ) cancer represents the sixth most frequent cause of cancer-related death globally and the eighth leading cancer diagnosed worldwide [1]. Over 90% of these tumors are squamous cell carcinomas (SCCs) [2]. 

The histological variants of SCC, which are included in the classification of the World Health Organization (WHO), independently by the anatomical location, are classic SCC, keratoacanthoma, acantholytic variant, spindle cell SCC, verrucous SCC and adenosquamous carcinoma. WHO also defined a group of uncommon variants, including SCC with sarcomatoid differentiation, lymphoepithelioma-like carcinoma and SCC with osteoclast-like giant cells. The aforementioned subtypes vary from well-differentiated, with intracellular or intercellular keratinization, to poorly differentiated and non-keratinized tumors [3]. Besides the histologic variants that are officially recognized by the latest WHO manual, SCC can also have a basaloid, clear cell or adenoid appearance [4].

In this paper, we present an extremely rare histological variant of the SCC of the GEJ. It is about the 24th case of SCC with signet-ring cell morphology described in the Medline database and the second case of the upper gastrointestinal tract. We describe the particularities of the case, from clinical management to molecular level, along with a review of the other 23 cases which were previously reported. A differential diagnosis with a clear cell variant is also presented.

## 2. Case Presentation

### 2.1. Personal History

A 59-year-old man was admitted to the General Surgery Department for recurrent pain and postprandial fullness in the epigastrium. The patient reported a 3-month history of the symptoms, which were accompanied by weight loss, lack of appetite, fatigability and constipation. He was a smoker of 20 cigarettes per day and a heavy consumer of alcohol on a daily basis. No family or personal history of oncologic diseases was known.

### 2.2. Laboratory Examinations

Hematological findings included microcytic hypochromic anemia, with a hematocrit of 34.3% (normal range 39–54%) and hemoglobin of 11.2 g/dL (normal range 13–17 g/dL); leukocytosis (13.77 × 10^3^ cells/µL; normal range 3.6–10 × 10^3^ cells/µL); and neutrophilia (11.64 × 10^3^ cells/µL; normal range 1.4–6.5 × 10^3^ cells/µL). Tumor markers, including carcinoembryonic antigen (CEA), carbohydrate antigen 125 (CA 125), carbohydrate antigen 19-9 (CA 19-9) and carbohydrate antigen 72-4 (CA 72-4), showed normal serum levels. The urine analysis revealed leukocyturia (75 cells/µL; normal value ≤ 20 cells/µL).

### 2.3. Imaging and Endoscopic Examinations

The preoperative computed tomography (CT scan) showed a heterogeneous, iodophilic, infiltrative thickening of the GEJ wall without the involvement of adjacent anatomical structures and without the suspicion of pulmonary or bone metastases. Upper digestive endoscopy confirmed the presence of a stenotic mass of the GEJ. The biopsy specimen was assessed as high-grade intraepithelial squamous dysplasia. As surgical resection was decided, the informed consent of the patient was obtained prior to surgery for the surgical intervention. The agreement of the Ethical Committee of the Clinical County Emergency Hospital, Targu Mures, Romania, was obtained for the processing and publication of the scientific data.

### 2.4. Surgery

Dissection of the para-esophageal soft tissue and fascia, followed by removal of the distal esophagus with part of the gastric fundus, was performed. The removed adenopathies were located near the common hepatic artery and left gastric artery and in the peri-esophageal space. The aforementioned adenopathies were excised and sent with the main specimen for histopathological assessment. 

### 2.5. Gross and Histopathological Assessment of Surgical Specimens

**Figure 1:**
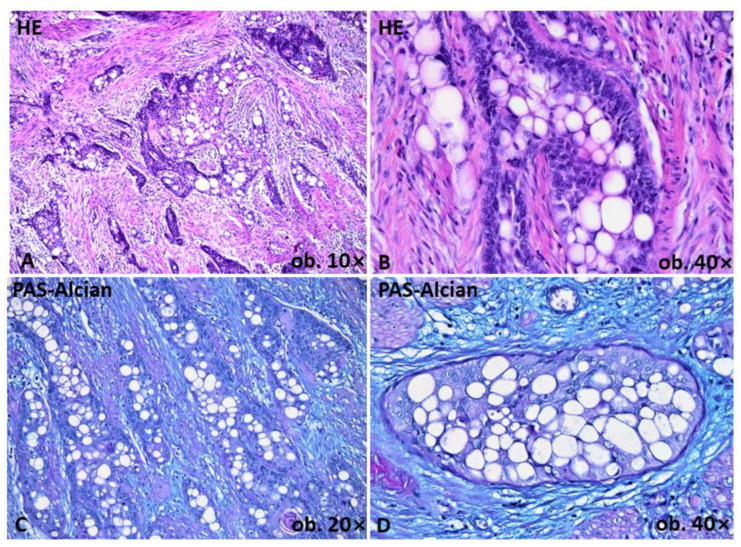
Histological features of the squamous cell carcinoma of the gastro-esophageal junction emphasized in Hematoxylin-Eosin and Periodic Acid Schiff and Blue Alcian (PAS-Alcian). Squamous nests are admixed with cells with signet-ring appearance in over 30% of tumor (**A**,**B**). No mucin or glycogen content is revealed at PAS-Alcian stain (**C**,**D**).

Macroscopically, a 33 × 30 mm ulcerative tumor mass of the GEJ, with a maximum thickness of 23 mm, was confirmed to induce luminal stenosis. On microscopic examination, in situ SCC was observed at the periphery of the tumor. The infiltrative mass crossed the circumferential resection margin and was mainly composed of squamous cells. Besides the squamous component, over 30% of the tumor cells showed a signet-ring cell appearance, with peripherally displaced nuclei, in a crescentic fashion, due to vacuolation of the cytoplasm. Signet-ring cells demonstrated no mucus or glycogen production with the application of periodic acid Schiff and Alcian blue stains (Figure 1). No histological signs of Barrett’s esophagus were seen. Out of the 13 regional lymph nodes, 2 presented metastases. Based on these aspects, the tumor was considered a pT3N1-SCC with a signet-ring cell component.

### 2.6. Immunohistochemical and Molecular Profile of Tumor Cells

**Figure 2:**
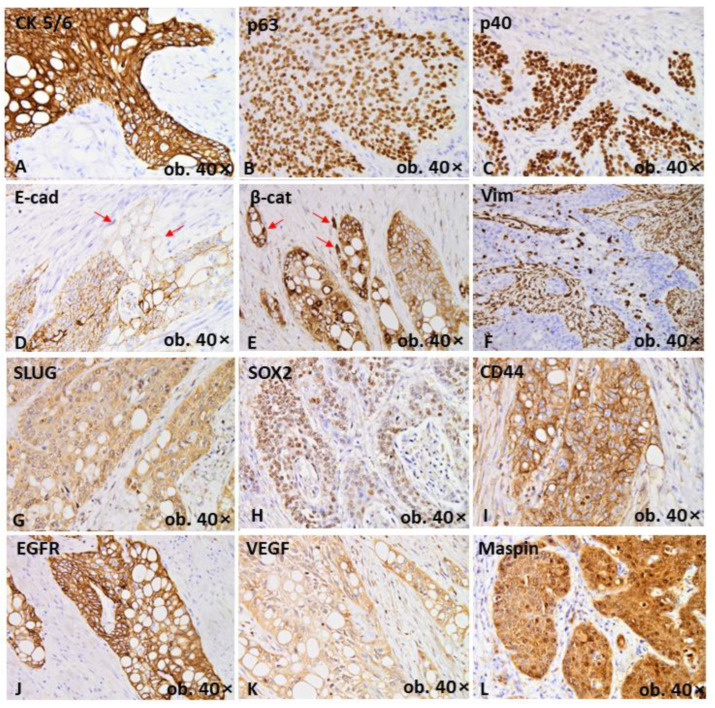
Tumor immunoprofile, confirming the diagnosis of SCC, with positivity of three markers of squamous differentiation—cytokeratin 5/6 (**A**), p63 (**B**) and p40 (**C**). Solid component keeps an epithelial phenotype, while signet-ring cells exhibit epithelial-mesenchymal transition emphasized by focal loss of E-cadherin-red arrows (**D**), nuclear translocation of β-catenin-red arrows (**E**) and positivity for vimentin (**F**) and Slug (**G**). Stemness capacity of tumor cells is emphasized by Sox2 (**H**) and CD44 (**I**). Diffuse positivity for EGFR (**J**) and VEGF (**K**) can also be seen, same as mixed nuclear-cytoplasmic maspin expression (**L**).

Based on the immunohistochemical profile, which was assessed for the conventional and signet-ring cell components, it was concluded that the signet-ring cells exhibited squamous differentiation. They showed positivity for keratin 5/6, p63 and p40. Mismatch repair (MMR) proficient/microsatellite stable (MSS) status was confirmed by the presence of diffuse nuclear positivity for all markers used to check the MMR profile (MLH1, MSH2, MSH6 and PMS2) in both components. EGFR (epidermal growth factor receptor), VEGF (vascular endothelial growth factor) and maspin showed diffuse positivity (Figure 2). No positivity was proved for CD68, CEA, CA 125, CA 19-9, CA 72-4, p16, PD-1 (programmed death 1), PD-L1 (programmed death-ligand 1) or Oct3/4 (Table 1). The in situ component demonstrated nuclear positivity for Epstein–Barr virus (EBV), but the infiltrative component was negative.

A dual population was revealed, which was considered evidence of epithelial–mesenchymal transition (EMT). EMT is defined as a partial or total loss of E-cadherin positivity, along with positivity for mesenchymal markers such as Slug and vimentin and membrane-to-nuclear translocation of β-catenin [5]. The conventional SCC cells retained diffuse membrane positivity for E-cadherin and β-catenin, demonstrating an epithelial molecular subtype. Meanwhile, the signet-ring cells demonstrated a mesenchymal phenotype, with partial loss of E-cadherin, nuclear translocation of β-catenin and diffuse positivity for Slug and vimentin (Figure 2). Both components were negative for N-cadherin, as well as HER-2 and V-set and Immunoglobulin domain containing 1 (VSIG1), a cell–cell adhesion considered specific for gastric adenocarcinoma [5]. 

The stemness capacity of tumor cells was checked with Sox2 and CD44. Sox2 showed nuclear positivity only in solid nests, with a loss of positivity in signet-ring cells, while CD44 showed positivity in both tumor populations (Figure 2). 

Molecular analysis demonstrated no mutation of *BRAF* (V600E) and *KRAS* genes (codons 12 and 13). Gene analysis was performed with qPCR, using in-vitro diagnosis (IVD) *BRAF* and *Kras* kit (Qiagen, Venlo, The Netherlands), based on scorpion primers. 

### 2.7. Outcome and Follow-Up

The oncologist decided on a standard regimen of chemotherapy with cisplatin and fluorouracil, combined with radiotherapy at a total dose of 50.4 Gy. Thirty-one months after surgery, at the last periodic follow-up, the patient was disease-free, with no local recurrence and no known distant metastases.

## 3. Discussion

SCC with a signet-ring cell component is an extremely rare morphologic variant, with only 24 cases reported in the Medline and Web of Science databases, including the present patient. Most of the cases were reported as affecting the external surface of the body, the uterine cervix, the lung or the oral cavity [6,7,8,9,10,11,12,13,14,15,16,17,18,19,20,21,22,23,24] (Table 2). To the best of our knowledge, this is the first case of SCC with a major component of signet-ring cell morphology affecting the GEJ. A similar case was reported in 2016 by Lee, but the signet-ring morphology was only isolated, with osteoclast-like individual and isolated giant cells, on a background of a predominant acantholytic squamous cell esophageal carcinoma [10].

For a long time, the signet-ring cell morphology was thought to be restricted to adenocarcinomas. Later, the signet-ring cell component was reported in SCC, mesothelioma, urothelial carcinoma and lymphoma, and even in mesenchymal tumors [8]. The reports based on histochemical findings postulated that the cellular cytoplasmic changes represent mucin, lipidic, glycogenic or even immunoglobulin storage [8,11]. However, even with electron microscopy, the nature of the vacuoles could not be definitively revealed. In the skin, the enlargement of rough endoplasmic reticulum cisternae was postulated to be present [7]. In our case, mucin and glycogen were excluded as contents of the vacuoles with periodic acid Schiff and Alcian blue staining.

The lower esophagus is predominantly the site of adenocarcinoma and not SCC, which mostly affects the upper two-thirds of the esophagus [4]. Therefore, adenosquamous carcinoma should first be ruled out as a differential diagnosis [4]. A challenging aspect is the differentiation of the signet-ring cell component from the clear cell and acantholytic/adenoid SCC. The signet-ring cell and clear cell variants cannot be differentiated using immunohistochemistry or special stains, both having clear cytoplasm. Therefore, the location of the nucleus—eccentric in the former and central in the latter—helps to differentiate the two variants. Conversely, the acantholytic variant not only shows a different architecture, with tumor nests with central acantholysis, often with giant, bizarre cells, but it also expresses, at least focally, CEA, which was absent in our case (Table 3). Acantholytic/adenoid tumors can also show loss of E-cadherin expression in the discohesive component [10].

Regarding the biphenotypic differentiation, the present case suggests the role of the Wnt/β-catenin-signaling pathway in the EMT of the SCC cells, which transition might induce transformation in cells with a signet-ring appearance. If the EMT phenomenon might be responsible for the behavior of the signet-ring cell component, the stemness of tumor cells might induce the aggressivity of the conventional SCC. In the present case, the nuclear transcription factor Sox2, known as a stem cell marker [21,22], showed nuclear positivity in the solid component only. From these facts, it can be supposed that the stemness features of non-signet-ring tumor cells might precede the development of mesenchymal status, with subsequent transformation into pseudo-signet-ring cells. Once the mesenchymal status is achieved, the stemness features, no longer needed, are lost, according to Sox2 expression. Liu et al., in 2018, also showed that upregulation of Sox2 led to overexpression of EMT markers such as vimentin, Twist, Snail and Slug, with subsequent reduction in E-cadherin membranous expression [23], similar to the present case. Conversely, other studies, such as that published by Bayo et al., demonstrated that tumor aggressivity was enhanced, contrary to Liu et al.’s research, by the loss of Sox2 expression, via upregulation of vimentin and subsequent motility acquirement [23,25]. While Liu et al. demonstrated the overexpression of EMT markers on the same cells that showed upregulation of Sox2 [23], our study emphasizes the idea that Sox2-expressing cells represent the precursors of those that will undergo EMT.

Because of the extremely low number of reports on such cases, the clinical course and prognosis of this particular SCC subtype are not fully known. Although the extreme aggressiveness of signet-ring morphology in glandular tumors is well known, in the present case, the patient had 31 months of disease-free survival at the most recent follow-up. This fact might be an indicator that the molecular changes responsible for signet-ring cell features differ between adenocarcinoma and SCC. The case affecting the lung, reported by Yigit et al. in 2018, was disease-free at 9 months after tumor excision [8]. Further, regarding the tumors affecting the skin, no differences in survival rates were observed between this variant and the classic types [12].

## 4. Conclusions

This case report highlights the complex implications of both EMT and the acquisition of stemness features of a biphenotypic SCC of the GEJ in enhancing tumor aggressivity and emphasizes the better specificity of Sox2 as a stem cell marker compared with CD44. Understanding the molecular background of the rare histologic subtypes might have therapeutic implications.

## Figures and Tables

**Table 1:** Immunohistochemical panel used to confirm the diagnosis of squamous cell carcinoma with signet-ring cell component and to emphasize the particular molecular features and stemness of the two tumor components.

Antibody	Clone	Manufacturer/ Country	Retrieval	Dilution	Classic SCC Component	Signet-Ring Cells
Cytokeratin	AE1/AE3	ImmunoLogic/ Netherland	High pH	RTU	Positive	Positive
Keratin 5/6	D5/16B4	Dako/Denmark	High pH	RTU	Positive	Positive
p63	4A4	ImmunoLogic	High pH	1:100	Positive	Positive
p40	ZR8	BioSB/USA	High pH	1:50	Positive	Positive
MLH1	ES05	Dako	High pH	RTU	Positive	Positive
MSH2	FE11	Dako	High pH	RTU	Positive	Positive
MSH6	EP49	Dako	High pH	RTU	Positive	Positive
PMS2	EP51	Dako	High pH	RTU	Positive	Positive
β-catenin	β-catenin1	Dako	High pH	RTU	Membrane positivity	Nuclear positivity
E-cadherin	NCH-38	Dako	High pH	RTU	Positive	Focally loss
N-cadherin	6G11	Dako	Citrate	1:100	Negative	Negative
Vimentin	V9	Dako	High pH	1:800	Negative	Positive
Slug	H-140	Santa Cruz/USA	High pH	1:100	Negative	Positive
HER2	Polyclonal	Dako	High pH	1:800	Negative	Negative
PD-1	NAT105	A.Menarini/Italy	High pH	1:50	Negative	Negative
PD-L1	22C3	Dako	Citrate	1:50	Negative	Negative
VEGF	VG-1	Abcam/UK	High pH	1:50	Negative	Negative
EGFR/HER1	H11	Dako	High pH	1:100	Positive	Positive
Sox2	Polyclonal	EMD Millipore/USA	Citrate	1:6000	Negative	Positive
Oct3/4	N1NK	Dako	High pH	1:50	Negative	Negative
CD44	DF1485	Dako	Citrate	1:50	Positive	Positive
CEA	II-7	Dako	High pH	1:50	Negative	Negative
CA125	M11	Dako	Citrate	1:20	Negative	Negative
CA19-9	CA19-9	Leica/UK	Citrate	1:200	Negative	Negative
CA72-4	B72.3	ThermoScientific/ UK	High pH	1:200	Negative	Negative
EBV	CS.1-4	Dako	Citrate	1:100	Negative	Negative
p16	Anti-p16	Biotech/USA	High pH	1:100	Negative	Negative

CA—carbohydrate antigen; CD—cluster of differentiation; CEA—carcinoembryonic antigen; EBV—Epstein Barr virus; EGFR—epidermal growth factor receptor; HER—human epidermal growth factor receptor; mb—membrane; MLH—MutL homolog; MSH—MutS homolog; Oct—octamer; PD—programmed death; PD-L—programmed death-ligand; PMS—postmeiotic segregation; RTU—ready to use; Sox—sry-related HMG-box.

**Table 2:** Cases of SCC with signet-ring cell morphology reported up to date in the Medline database.

Case No.	Authors, Year of Publication	Patient’s Gender	Patient’s Age (Years)	Localization	Diameter (mm)
1.	Cramer and Heggeness (1988) [6]	Male	69	Skin (forehead)	NA
2.	Kupryjanczyk et al. (1992) [9]	Female	74	Uterine cervix	NA
3.		Female	84	Uterine cervix	NA
4.	McKinley et al. (1998) [7]	Male	50	Skin (neck)	6 mm
5.	Bastian et al. (1999) [15]	Female	79	Skin (right cheek)	NA
6.	Bastian et al. (1999) [15]	Male	82	Skin (temple)	NA
7.	Bastian et al. (1999) [15]	Male	83	Skin (ear)	NA
8.	Bastian et al. (1999) [15]	Male	80	Skin (forehead)	NA
9.	Bastian et al. (1999) [15]	Male	87	Skin (scalp)	NA
10.	Bastian et al. (1999) [15]	-	-	-	-
11.	Bastian et al. (1999) [15]	Male	76	Skin (forehead)	NA
12.	Proia et al. (2006) [16]	Female	93	Skin (eyelid)	17 mm
13.	El Demellawy et al. (2011) [12]	Female	84	Upper Lip	11 mm
14.	Lortscher et al. (2012) [17]	Male	67	Skin (canthus/palpebral commissure)	15 mm
15.	Nakajima et al. (2013) [11]	Male	83	Skin (finger)	31 mm
16.	Park (2015) [13]	Male	64	Lung	29 mm
17.	Wang et al. (2016) [14]	Female	78	Skin (thigh)	50 mm
18.	Lee (2016) [10]	Female	82	Esophagus	55 mm
19.	Yigit et al. (2018) [8]	Male	85	Lung	42 mm
20.	Handra-Luca (2019) [18]	-	-	Lung	-
21.	Findeis et al. (2020) [19]	Female Transgender	66	Skin (parotid region)	56 mm
22.	Haghayeghi et al. (2020) [20]	Male	71	Skin (ear)	15 mm
23.	Apandi et al. (2022) [24]	Male	65	Oral cavity (gingiva)	10 mm
24.	Present case	Male	59	Esophagus	33 mm

**Table 3:** The differential diagnosis criteria for three particular histologic subtypes of squamous cell carcinoma with similar features.

	Signet-Ring SCC	Clear Cell SCC	Acantholytic/Adenoid/ Pseudo-Glandular SCC
Histology	→ peripherally displaced nucleus → large cytoplasmic vacuoles → often with a second component of classic SCC	→ centrally located nucleus → hydropic cytoplasm → type I-keratinizing → type II-nonkeratinizing → type III-pleomorphic	→ discohesive acantholytic cells → bizarre, often multinucleated cells
Special stains	→ PAS-negative → Blue Alcian-negative → Mucicarmine-negative	→ PAS-negative → Blue Alcian-negative → Mucicarmine-negative	→ PAS-neg → Blue Alcian-negative → Mucicarmine-negative
IHC	→ keratin AE1/AE3 + → keratin 5/6 + → P63 + → P40 + → CEA –	→ keratin AE1/AE3 + → keratin 5/6 + → P63 + → P40 + → CEA –	→ keratin AE1/AE3 + → keratin 5/6 + → P63 + → P40 + → CEA + → EMA +
Clinical behavior	High aggressivity	Moderate/high aggressivity	Moderate aggressivity

CEA—carcinoembryonic antigen.

## Data Availability

The details of the case are available at the corresponding authors upon request.
